# Video Consultations for Patients Traveling Internationally for Medical Care: An Observational Study of a Tertiary Hospital in South Korea

**DOI:** 10.3390/ijerph22040481

**Published:** 2025-03-24

**Authors:** Mirim Byun, Hyun-Ju Baek, Taeseo Kim, Jaehyun Jung, Hyelim Lee, Yoojung Lee, Sergey Kim, Wonjae Lee, Sung Hee Choi, Seung-Yeon Lee, Ji Soo Kim

**Affiliations:** 1 International Healthcare Center, Seoul National University Bundang Hospital, Seongnam-si 13620, Republic of Korea; 11596@snubh.org (M.B.); hjbaek@snubh.org (H.-J.B.); taeseo1104@snubh.org (T.K.); jhyun@snubh.org (J.J.); hr72@snubh.org (H.L.); michelle@snubh.org (Y.L.); kimsv@snubh.org (S.K.); wpiglet7@snubh.org (W.L.); drshchoi@snubh.org (S.H.C.); leesy8503@snubh.org (S.-Y.L.); 2 Division of Cardiology, Department of Internal Medicine, Seoul National University Bundang Hospital, Seongnam-si 13620, Republic of Korea; 3 Division of Endocrinology, Department of Internal Medicine, Seoul National University Bundang Hospital, Seongnam-si 13620, Republic of Korea; 4 Department of Family Medicine, Seoul National University Bundang Hospital, Seongnam-si 13620, Republic of Korea

**Keywords:** medical tourism, international medical travel, online healthcare, video consultation

## Abstract

With the rise in patients traveling worldwide for medical treatment in recent years, the importance of patient care continuum has become increasingly evident. We aimed to analyze the role of video consultations in facilitating medical care for patients traveling internationally for medical care in South Korea. In this retrospective study, data were gathered on video consultations for international medical travelers conducted between January 2021 and December 2023 at a single tertiary hospital. We analyzed information on patient demographics, the content of video consultation, and how these factors were associated with the patient’s decision to travel for treatment. A total of 16.5% of international medical travel patients who underwent video consultation subsequently traveled for treatment. Patients who engaged directly with the institution were more likely (aOR 5.74, [95% CI 1.20–27.33]) to travel for treatment compared to those who relied on domestic or international travel services. Additionally, patients who participated in follow-up video consultations were more likely (aOR 4.06, [95% CI 1.04–15.92]) to travel compared to those who underwent their first video consultation. International medical travel patients utilized video consultations for a range of medical conditions, with the likelihood of subsequent treatment travel being associated with both the medium through which consultations were initiated and prior experience with the institution.

## 1. Introduction

International tourism experienced a significant resurgence in 2023, indicating a strong potential to return to pre-pandemic levels by 2024 [1]. Reflecting this trend, South Korea has emerged as a prominent destination for medical tourists, with a cumulative total of 3,271,574 patients by 2022 [2]. Telemedicine has increasingly been recognized as an invaluable tool, particularly in the aftermath of the COVID-19 pandemic, for providing specialized medical guidance, especially to individuals facing health challenges while traveling to distant locales [3]. It plays a crucial role in medical tourism by reducing costs, improving access to specialized healthcare, ensuring continuity of care for international patients, expanding the market, and enhancing the reputation of medical institutions [4,5]. Kothari et al. highlights that telemedicine can enhance quality, efficiency, and customer service in medical tourism by improving coordination between healthcare providers, as well as optimizing preoperative, postoperative care, and travel for patients and their families [6]. In effect, telemedicine can reduce travel costs and time for patients, provide clear treatment plans through pre-consultations, and offer personalized care, thereby enhancing the efficiency of medical tourism and improving patient satisfaction.

Despite the acknowledged potential of telemedicine, research on its application for international medical travel patients remains severely limited. Previous telemedicine research consists of reviews and predominantly focuses on providing healthcare for nationals. For example, a study by Rochat and Genton examined the use of teleconsultation in travel clinics for domestic residents traveling abroad [3]. Other studies, such as that of Hernández, have explored the medical and economic benefits of telemedicine within a domestic region [7]. In South Korea, Article 16 of the ‘Act on Support for Medical Overseas Expansion and Attraction of Foreign Patients’ [8] permits telemedicine between medical professionals, but does not explicitly allow remote consultations between doctors and patients, limiting the broader use of telemedicine [9]. While telemedicine services were temporarily expanded for domestic patients during the COVID-19 pandemic [10], there is still no permanent legal framework to integrate telemedicine into medical tourism [11].

This paper aims to comprehensively analyze actual cases of video consultations conducted over three years (2021–2023) among foreign patients at a single tertiary hospital in South Korea. This study seeks to identify the demographic characteristics of the patients, determine any associations between consultation content and subsequent patient travel for treatment, and delineate the role of video consultation as a tool for international medical travel patients.

## 2. Materials and Methods

### 2.1. Video Consultation Process 

**Figure 1:**
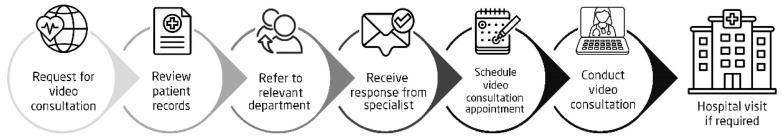
Patient workflow of video consultation.

The process of conducting a video consultation follows a structured sequence (Figure 1). It begins with a request for consultation from patients worldwide. Following a thorough examination of patient records by both the attending physician and coordinator at the international healthcare center to ensure a comprehensive understanding of the patient’s medical history, relevant information is uploaded to the Electronic Medical Record (EMR) system. Notably, Seoul National University Bundang Hospital (SNUBH) has been utilizing its self-made EMR system, BESTCare 2.0 (ezCaretech, Seoul, South Korea), since its establishment in 2003 [12]. Subsequently, referrals are made to the relevant specialist based on the examination of patient records. Specialists provide their insights and opinions via the EMR system. Upon receiving the specialist’s response, an appointment for the video consultation is scheduled, considering the availability of both parties.

In 2021, SNUBH launched a hospital EMR-based video consultation platform named ezOntheCall. This platform, integrated with BESTCare 2.0, allows for seamless and continuous patient care management. In cases where further evaluation or treatment is deemed necessary, arrangements for a hospital visit are made. To ensure accurate cost estimates, at least three cases of Korean patients with similar conditions within the past five years are reviewed to calculate an average cost (estimated cost of treatment). This structured approach ensures efficient and effective video consultation, leveraging advanced technology to provide comprehensive healthcare services to patients, regardless of geographical constraints.

### 2.2. Data Collection

The data for this study were collected from the hospital EMR system (BESTCare 2.0) and anonymized video consultation logs at SNUBH. The focus was on foreign patients without Korean national insurance who participated in video consultations from 1 January 2021 to 31 December 2023. Collected variables encompassed demographic details such as age, gender, and nationality, as well as clinical information including diagnosis and the medium through which consultations were requested, alongside the estimated cost of treatment.

Regional classifications were determined as follows: Commonwealth of Independent States (CIS) countries (Russia, Uzbekistan, Kazakhstan, Georgia), non-CIS Asia (Mongolia, Cambodia), North America (U.S.), and the Middle East (UAE). According to the 2022 Foreign Patient Attraction Performance Statistics Report, these regions account for a significant proportion of inbound medical travelers to South Korea, aligning with the patient distribution at our institution [2]. CIS countries were grouped together, as they are members of or closely aligned with the CIS, reflecting shared historical, cultural, and economic ties, as well as similarities in healthcare infrastructure and medical travel trends. Mongolia and Cambodia were classified together as a non-CIS Asia group. Medical travelers from this group show a strong preference for South Korea’s healthcare system due to factors such as geographical proximity, high levels of trust in Korean medical services, and the significant volume of medical travel, particularly from Mongolia. Patients from North America, where highly developed healthcare systems are available but outbound medical tourism remains significant, often travel for cost-effective, specialized, or timely medical care in South Korea. This category also includes expats and U.S. military patients residing in South Korea. Middle Eastern countries were grouped together due to their geographical proximity and their active engagement in international medical travel. These nations have high healthcare expenditures and frequently seek advanced medical treatments abroad.

Data collection involved categorizing video consultation requests based on the source of referral (request medium). The term direct refers to cases where patients contacted the facility directly—such as through hospital email, website, or social media—without the involvement of intermediary agencies that recommend patients to other countries for medical treatment. On the other hand, third party encompasses cases where referrals were facilitated by intermediary agencies. Third-party referrals were further categorized into domestic and international parties. Domestic refers to agencies registered within Korea and internationally, agencies in countries other than Korea. In this study, the term initial refers to the first video consultation with SNUBH that occurred without an in-person hospital visit. Follow-up includes patients who initially received treatment at the hospital and continued their care through subsequent video consultations, as well as those who began with an initial video consultation and proceeded with additional video consultations thereafter. Cases were categorized as surgical, oncological, or others, based on the perceived severity of the condition and the prioritization of medical attention required. Cases requiring surgery were categorized as surgical. Oncological cases were categorized to include both benign and malignant conditions. For benign cases without diagnostic confirmation, video consultations were conducted under the assumption of potential malignancy. All other conditions were categorized as other.

### 2.3. Statistical Analyses

Participants were evaluated for adjusted odds ratios (aORs) and 95% confidence intervals (95% CIs) of medical travel using multivariate logistic regression after adjustments for the covariates. The considered covariates included age (categorical, 0–19, 20–39, 40–59, 60 and above), sex (categorical, male, female), nationality (categorical, CIS countries, non-CIS Asia, North America, Middle East), year of video consultation (categorical, 2021, 2022, 2023), consultation category (categorical, initial, follow-up), medium used for video consultation request (categorical, third party (domestic)), third party (international, direct), and diagnosis at the time of video consultation (categorical, surgical, oncological, other). Statistical significance was defined as *p*-value < 0.05. *p*-value by Chi-square test for categorical variables was used to determine the association with medical travel. Statistical analyses were conducted using STATA/SE ver. 16.0 (Stata Corp., College Station, TX, USA).

### 2.4. Ethics

The present study protocol was reviewed and approved by the Institutional Review Board (IRB) of Seoul National University Bundang Hospital (IRB No. B-2407-911-103). Informed consent was waived because of the retrospective nature of the study.

## 3. Results

The general characteristics of the study population, categorized by whether individuals proceeded with medical travel following video consultation or not, are presented in Table 1. Out of the total 182 patients who underwent video consultation, 30 (16.5%) opted for medical travel for in-person treatment (referred to as “Travelers”), while 152 (83.5%) did not (referred to as “Non-travelers”).

In the “Travelers” group, males were more prevalent compared to females, whereas in the “Non-travelers” group, females were more prevalent than males. Surgical cases constituted a higher proportion of consultations in the “Travelers” group. Both groups predominantly comprised individuals aged 40–59 years.

Patients originating from CIS countries such as Russia, Uzbekistan, and Kazakhstan, along with non-CIS Asian countries, represented the largest proportions in both the “Travelers” and “Non-travelers” groups. The majority of video consultation requests were facilitated through the domestic third-party channel. Throughout the study period, the year 2022 witnessed the highest number of consultations.

Table 2 presents the results of a multivariable logistic regression analysis investigating factors associated with video consultation leading to medical travel. Patients who underwent video consultation directly through personal contact were more likely to pursue medical travel following the consultation compared to those who utilized domestic third-party services (aOR 5.74, [95% CI 1.20–27.33]). Compared to the initial consultation group, the follow-up consultation group was more likely to choose medical travel after video consultation (aOR 4.06, [95% CI 1.04–15.92]).

Table S1 presents factors related to medical travelers, including the time interval from video consultation to arrival and the pre-provided estimated cost for treatment for patients. Among these travelers, 36.7% traveled within 30 days of the consultation, while 63.3% traveled after this period. 46.7% had estimated treatment costs below USD 5000, 6.7% had costs between USD 5000 and 10,000, and 46.7% exceeded USD 10,000 in estimated expenses.

## 4. Discussion

Based on data collected from a single tertiary hospital in South Korea, our study found that 16.5% of international medical travel patients who underwent video consultation subsequently traveled for treatment. Notably, patients who engaged directly with the institution or participated in follow-up video consultations were more likely to travel compared to those who utilized third-party services or underwent their initial consultation, respectively. To our knowledge, this is the first study to examine the use of video consultation in international medical travel patients.

The significance and advantages of pre- and post-travel consultations have been studied [13,14]. However, existing studies on medical tourism have primarily focused on patient experiences or the industry perspective [15,16]. Telemedicine, as a tool for consultations, not only facilitates coordination between primary care physicians and specialists but also enhances health outcomes by ensuring a care continuum [17] and potentially minimizes cost [7,18]. Consistent with earlier findings, our results indicated that individuals aged 40–59 years comprised the majority of those who utilized video consultation [19]. Kruse et al. also identified age as a factor influencing telemedicine adoption, suggesting that utilization patterns may vary across different age groups [20]. Furthermore, patients originating from CIS countries represented the largest demographic among those who underwent and traveled after video consultation. This reinforces the idea that both cultural and geographical distance have an impact on patients’ choice of medical treatment destinations [21].

The decision-making process for patients considering travel for medical care remains complex [22]. Our findings reveal that individuals who directly contacted the institution were 5.8 times more likely to travel for treatment compared to those who relied on domestic or international travel services. This highlights the crucial role of information source as a vital antecedent of shaping destination perception and intention [23]. Additionally, patients who underwent video consultation for follow-up evaluation were 4.23 times more likely to travel for treatment, underscoring the value of experiences from previous video consultation and/or visit to the institution, with implications to the role of video consultation for enhancing continuity of care.

Our analysis included a diverse range of medical cases, from infectious to chronic and serious conditions (Table S2). While surgical cases were the most common reason for video consultation, patients requiring oncological treatment were more likely to travel in comparison to those requiring surgery (statistically not significant) [24]. These findings align with a prior study conducted at a travel clinic, which demonstrated that pre-travelers, particularly those who are immunosuppressed, showed interest in telemedicine via emails [3]; however, the focus of the study was not on medical travel or inbound patients. The application of telemedicine to cancer care has been reviewed previously [25], and further investigation is warranted to elucidate the effect of video consultation on clinical outcomes.

This study has several limitations. First, video consultation cases were confined to a single institution, limiting the scope of analysis, and outcomes for patients who did not receive video consultations were not observed. Second, potential biases may have emerged from the lack of adjustment for the duration of video consultations and pre-provided estimated treatment costs. Third, we did not differentiate between follow-up consultation patients who had previously visited the institution and those who had multiple video consultations. In addition, the role of video consultation based on clinical outcomes of the care continuum was not observed. Finally, we did not collect data on patient or physician experiences following video consultations. According to post-treatment satisfaction surveys conducted on patients who traveled for care, responses regarding pre-travel treatment planning were predominantly positive, whereas satisfaction ratings related to treatment costs showed variability (data not presented).

## 5. Conclusions

In conclusion, our findings indicate that in the decision-making process of traveling for treatment, patients traveling internationally for medical care utilized video consultations for a range of medical conditions, with the likelihood of subsequent treatment travel being associated with both the medium through which consultations were initiated and prior experiences with the institution. There is a pressing need for the development and incorporation of user-friendly, sustainable platforms for medical consultation requests as well as structured pre- and post-travel consultations.

## Figures and Tables

**Table 1:** General characteristics of patients who underwent video consultation.

Characteristic	Patients Who Underwent Video Consultation N = 182	
	Non-Travelers N = 152 (83.5%)	Travelers N = 30 (16.5%)
Gender		
Male	71 (46.7)	18 (60.0)
Female	81 (53.3)	12 (40.0)
Age		
0–19	31 (20.4)	4 (13.3)
20–39	38 (25.0)	8 (26.7)
40–59	47 (30.9)	14 (46.7)
≥60	36 (23.7)	4 (13.3)
Disease/Specialty		
Surgical	53 (34.9)	14 (46.7)
Oncological	41 (27.0)	8 (26.7)
Other	58 (38.2)	8 (26.7)
Region		
CIS countries	61 (40.1)	14 (46.7)
Non-CIS Asia	89 (58.6)	12 (40.0)
North America	1 (0.7)	3 (10.0)
Middle East	1 (0.7)	1 (3.3)
Request Medium		
Third-party (domestic)	108 (71.1)	13 (43.3)
Third-party (international)	23 (15.1)	8 (26.7)
Direct	21 (13.8)	9 (30.0)
Year		
2021	56 (36.8)	11 (36.7)
2022	75 (49.3)	15 (50.0)
2023	21 (13.8)	4 (13.3)

**Table 2:** Factors associated with video consultation leading to medical travel: multivariable logistic regression analysis.

Characteristic	OR (95% CI)	*p*-Value
Gender		
Male	1	
Female	0.66 (0.27–1.63)	0.366
Age		
0–19	1	
20–39	1.72 (0.38–7.65)	0.479
40–59	2.80 (0.67–11.68)	0.157
≥60	0.46 (0.07–2.99)	0.414
Disease/Specialty		
Surgical	1	
Oncological	2.12 (0.53–8.53)	0.290
Other	0.87 (0.20–3.71)	0.851
Region		
CIS countries	1	
Non-CIS Asia	0.99 (0.25–3.85)	0.987
North America	12.97 (0.77–220.00)	0.076
Middle East	5.43 (0.03–981.71)	0.524
Request Medium		
Third-party (domestic)	1	
Third-party (international)	3.54 (0.82–15.32)	0.091
Direct	5.74 (1.20–27.33)	0.028
Video Consultation Category		
Initial	1	
Follow-up	4.06 (1.04–15.92)	0.044
Year		
2021	1	
2022	1.59 (0.54–4.67)	0.397
2023	0.35 (0.08–1.60)	0.175

Adjusted for age, sex, nationality, year, consultation category, request medium, and diagnosis.

## Data Availability

The datasets used are available from the corresponding author upon reasonable request.

## Supplementary Materials

The following supporting information can be downloaded at https://www.mdpi.com/article/10.3390/ijerph22040481/s1, Table S1: The time interval from video consultation to arrival and the pre-provided estimated cost for patients who underwent medical travel following video consultation; Table S2: ICD-10 disease classification and corresponding codes for patients who underwent medical travel following video consultation.

## Abbreviations

The following abbreviations are used in this manuscript:

SNUBH	Seoul National University Bundang Hospital
EMR	Electronic Medical Record
CIS	Commonwealth of Independent States
