# Supplementary material for: Video Consultations for Patients Traveling Internationally for Medical Care: An Observational Study of a Tertiary Hospital in South Korea
Source: Int J Environ Res Public Health. 2025 Mar 24;22(4):481. doi: 10.3390/ijerph22040481 (PMC12026667; doi:10.3390/ijerph22040481)
Supplement: Supplementary file 1 [file ijerph-22-00481-s001.zip › ijerph-3481742-supplementary.pdf]

Table S1. **The time interval from video consultation to arrival and the pre-provided estimated cost for patients who underwent medical travel following video consultation**

**Video consultation-to-Travel-for-Treatment Time, N (%)**	
< 30 days	11 (36.7%)
≥ 30 days	19 (63.3%)
**Estimated Cost of Treatment, N (%)**	
< 5,000 USD	14 (46.7%)
≥5,000 , <10,000 USD	2 (6.7%)
≥ 10,000 USD	14 (46.7%)

Table S2. ICD-10 disease classification and corresponding codes for patients who underwent medical travel following video consultation

**ICD**	**Diagnosis**
C189	Colon cancer
C539	Cervical cancer
C719	Glioblastoma multiforme
C819	Hodgkin lymphoma
C840	Mycosis fungoides
C900	Multiple myeloma
D103	Benign neoplasm of soft palate
D141	Laryngeal papillomatosis
D169	Benign neoplasm of bone
D333	Vestibular schwannoma
D432	Brain tumor
E031	Congenital hypothyroidism
H46	Optic neuritis
H932	Hearing disorder
I251	Coronary disease
J342	Deviated nasal septum
K432	Incisional hernia
L709	Acne
M122	Pigmented villonodular synovitis
M411	Adolescent idiopathic scoliosis
M501	Herniated disc disease of cervical spine with radiculopathy
M541	Radiculopathy
M952	Cranial bone defect
M2116	Genu varum, acquired
M8795	Avascular necrosis of femoral head
Q070	Arnold-Chiari malformation with syringomyelia
